# Epidemiological Dynamics of Foot-and-Mouth Disease in the Horn of Africa: The Role of Virus Diversity and Animal Movement

**DOI:** 10.3390/v15040969

**Published:** 2023-04-14

**Authors:** Fanos Tadesse Woldemariyam, Christopher Kinyanjui Kariuki, Joseph Kamau, Annebel De Vleeschauwer, Kris De Clercq, David J. Lefebvre, Jan Paeshuyse

**Affiliations:** 1 Laboratory of Host-Pathogen Interaction in Livestock, Division of Animal and Human Health Engineering, Department of Biosystems, KU Leuven, 3001 Leuven, Belgium; christopherkinyanjui.kariuki@kuleuven.be; 2 College of Veterinary Medicine, Addis Ababa University, Bishoftu P.O. Box 34, Ethiopia; 3 Institute of Primate Research, Karen, Nairobi P.O. Box 24481-00502, Kenya; jkamau@primateresearch.org; 4 Department of Biochemistry, University of Nairobi, Nairobi P.O. Box 30197, Kenya; 5 Sciensano, Service for Exotic and Vector-Borne Diseases, 1050 Brussels, Belgium

**Keywords:** foot-and-mouth disease, virus diversity, livestock movement, wild ungulates, Horn of Africa

## Abstract

The Horn of Africa is a large area of arid and semi-arid land, holding about 10% of the global and 40% of the entire African livestock population. The region’s livestock production system is mainly extensive and pastoralist. It faces countless problems, such as a shortage of pastures and watering points, poor access to veterinary services, and multiple endemic diseases like foot-and-mouth disease (FMD). Foot-and-mouth disease is one of the most economically important livestock diseases worldwide and is endemic in most developing countries. Within Africa, five of the seven serotypes of the FMD virus (FMDV) are described, but serotype C is not circulating anymore, a burden unseen anywhere in the world. The enormous genetic diversity of FMDV is favored by an error-prone RNA-dependent RNA polymerase, intra-typic and inter-typic recombination, as well as the quasi-species nature of the virus. This paper describes the epidemiological dynamics of foot-and-mouth disease in the Horn of Africa with regard to the serotypes and topotypes distribution of FMDV, the livestock production systems practiced, animal movement, the role of wildlife, and the epidemiological complexity of FMD. Within this review, outbreak investigation data and serological studies confirm the endemicity of the disease in the Horn of Africa. Multiple topotypes of FMDV are described in the literature as circulating in the region, with further evolution of virus diversity predicted. A large susceptible livestock population and the presence of wild ungulates are described as complicating the epidemiology of the disease. Further, the husbandry practices and legal and illegal trading of livestock and their products, coupled with poor biosecurity practices, are also reported to impact the spread of FMDV within and between countries in the region. The porosity of borders for pastoralist herders fuels the unregulated transboundary livestock trade. There are no systematic control strategies in the region except for sporadic vaccination with locally produced vaccines, while literature indicates that effective control measures should also consider virus diversity, livestock movements/biosecurity, transboundary trade, and the reduction of contact with wild, susceptible ungulates.

## 1. Search Strategy and Selection Criteria

Articles published in peer-reviewed journals were obtained via the online search engines: Pub Med, Google Scholar, and the World Reference laboratory resource for foot-and-mouth Disease (WMRLFMD) website (https://www.wrlfmd.org/ref-lab-reports). The language in which the search was performed was specified as English. The search items were limited to fully accessible manuscripts or reports. The time period for the literature to be retrieved electronically was set between 1 January 1999, and 9 March 2023.The key words used per the sections were as follows: **Introduction**: Livestock production system in East Africa OR Horn of Africa OR animal Product supply and demand Horn of Africa OR Livestock provides milk, meat, draft power OR Livestock Sector Report Horn of Africa OR FMD host range and pathogenesis OR FMD prevalence in the Horn of Africa OR Ethiopia OR Kenya OR Uganda OR Eritrea OR Sudan OR South Sudan. **Serotype or topotype diversity**: Serotype/topotype O or A OR SAT 1, 2,3 Horn of Africa OR Ethiopia OR Kenya OR Sudan OR South Sudan OR Eritrea OR Djibouti OR Uganda OR East Africa. **Acute and persistent infection**: Acute infection in cattle FMDV epidemiology OR Between animal and farm transmission of FMD OR FMD in carrier and persistent state OR Acute and persistent infection. **Livestock movement and trade**: Foot-and-mouth disease livestock movement OR pastoralism and livestock disease in East Africa OR foot-and-mouth disease virus dispersal in East Africa OR Cross border livestock movement in Africa. **Wild animals and FMDV**: Wild animals and foot-and-mouth disease in East Africa OR Role of wild ungulates for foot-and-mouth disease epidemiology OR Viral disease and African ungulates. **FMD vaccines**: Foot-and-mouth disease vaccines in East Africa OR Foot-and-mouth disease vaccines in Ethiopia OR Foot-and-mouth disease vaccine strain selection in East Africa. For the remaining sections, global FMD distribution OR FMD distribution, was used. In total, 210 articles were retrieved, and after applying the inclusion (serotypes, topotypes distribution in relation to animal movements, livestock production systems, and the role of wildlife in the horn of Africa) and exclusion (outside the horn area and not related to the mentioned factors) criteria, 102 citations were considered potentially eligible for inclusion in this review. In addition, for the most recent laboratory reports on the WRLFMD web site (search terms: world reference laboratory quarterly report, and the year), some articles were searched manually using the Google Search engine (search reference term: foot-and-mouth disease/epidemiology/prevalence/vaccines).

## 2. Introduction

The greater Horn of Africa, as part of East Africa, includes countries such as Djibouti, Ethiopia, Somalia, Eritrea, Kenya, Uganda, Sudan, and South Sudan. Approximately 40% of the African and 10% of the global livestock populations reside in this area [1].The total livestock population is estimated to be 532 million [2], and ruminants comprised 375 million, as reported by the Inter-governmental Authority for Development (IGAD) [3].The number of livestock in this region consists of 102, 104, 688 cattle, 11,742,390 camels, and 179, 579,520 small ruminants [4].

Foot-and-mouth disease (FMD) is one of the most important contagious livestock diseases and has an important economic impact globally. FMD affects over 70 domestic (e.g., cattle, sheep, goats, and swine) and wild (e.g., African buffalo, gazelle) cloven-hoofed animals [5] and exists in 7 serotypes (O, A, C, Asia1, SAT1, SAT2, and SAT3). The Horn of Africa hosts the FMDV serotypes O and A in order of prevalence [6]; the South African territories serotypes (SAT1 and SAT2) have been known to circulate in Ethiopia, Kenya, and Uganda as reviewed by [7]. In Uganda, SAT2 and SAT1 have been identified from the African Buffalo in the Queen Elizabeth National Park [8], and SAT3 was reported in Uganda in 2013 [9]. Serotype C has never been isolated from this region despite serological detection, and no clinical case of FMD caused by serotype C has been reported or detected over the last 15 years [10]. FMD is estimated to circulate in 77% of the livestock population, and the cost incurred as a result of this disease in Africa is 50% of the total cost of the continent [11]. Vaccination of a few dairy cattle herds is practiced but with limited success as the result of a lack of good quality vaccine (might be poor cold chain handling) or vaccination strategy, unrestricted animal movement, and poor biosafety/biosecurity measures [12,13]. In addition, investigations to determine matching vaccine strains are limited [14]. Mass vaccination, restriction of animal movement, and culling have never been practiced, at least in Ethiopia [15]. This review aims to highlight FMD dynamics in the greater Horn of Africa concerning the role of viral genetics and antigenic variability, trade and livestock movement, and the role of wildlife.

## 3. The Causes of FMD Virus Diversity

In general, the genetic diversity of ribonucleic acid (RNA) viruses such as FMDV results from the error-prone replication process of the RNA-dependent RNA polymerase (RdRp), large population diversity, and high replication rate [16] as well as recombination [17,18,19,20]. RNA viruses such as FMDV, without the involvement of any external driving force, use positive selection mechanisms termed broadly diversifying selection. This selection is built on a progressive increase in the number of different genomes, which in turn dominate the population [21]. High genetic diversity at the antigenic site or near the antigenic site can also be driven by a high substitution rate, purifying selection (negative selection), and diversifying selection (positive selection). This was identified on serotype A VP1, VP2, and VP3 capsid proteins. Since there is no error correction in foot-and-mouth disease by RdRp, the polymerase of RNA viruses, the error will go on to the next round of replication. Literature states that this low-fidelity polymerase of FMDV is one cause of genetic diversity, resulting in increased virulence and transmissibility of serotype O around the globe [22]. The competition within the quasi-species nature of FMDV is capable of generating a mutant variety at the level of the whole population [23,24].

In addition to the above, genetic recombination is another determinant in the generation of new variants. In FMDV 15, recombination events were more evident in the non-structural proteins than in the genes coding for the structural proteins (capsid) [18,19]. Further, experimental co-infection of cattle with serotypes A and O also confirmed the inter-serotype recombination of the virus in samples taken from the upper respiratory tract [25]. As an indication of the evolutionary interdependence of FMDV serotypes and topotypes, intra-lineage recombination of FMDV was reported [26].

## 4. Distribution of Foot-and-Mouth Disease

### 4.1. Global Distribution

Except for Greenland, Iceland, New Zealand, and the smaller islands of Oceania, every region in the world has, at least once, experienced an FMD outbreak. The outbreak reports of 1929, 1952, 1954, and 1970 in the USA, Canada, Mexico, and Australia, respectively, marked the last outbreak of FMD in these countries. The last sporadic outbreak of FMD on the European continent dates from 2011, as the disease was eradicated in Europe in the 1980s [27,28]. Globally, FMD has three continental epidemiological clusters, namely Africa, Asia, and South America. A significant number of sub-Saharan African and Asian countries are endemic to FMD. In the Americas, FMD can still be found in Venezuela.

**Figure 1:**
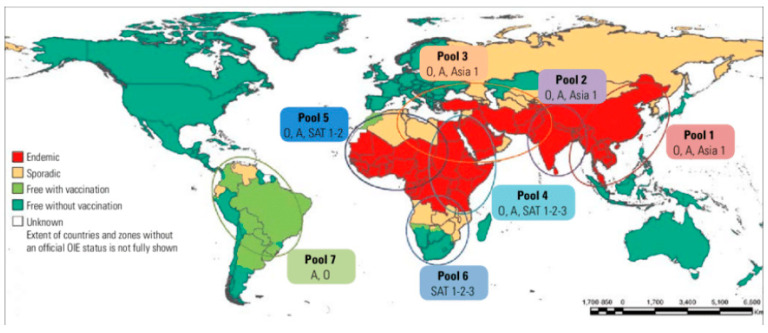
World map showing the distribution of foot-and-mouth disease (FMD) virus pools by serotype (source:) (reprinted and Adapted from [37].

Globally, FMD is distributed into seven virus pools, and within each pool, several serotypes of FMDV can be found [29,30]. Africa, Asia, and South America historically hold six (i.e., A, O, C, SAT1, SAT2, and SAT3), four (i.e., A, O, Asia1, C), and three (i.e., A, O, C) serotypes, respectively. In Africa, pool 4 covers Eastern Africa, pool 5 covers Western Africa, and pool 6 covers Southern Africa. Pools 4 and 5 are endemic for the FMDV serotypes O, A, SAT1, and SAT2. Serotype C was reported for the last time in Kenya in 2004 [31,32] and reviewed by Di Nardo et al. [33]. Serologically, the presence of serotype C has been reported in Eritrea [34] and in the Borena zone of southern Ethiopia [35]. To rule out the presence or absence of this serotype, further investigation needs to be performed as recommended [36]. Serotypes SAT1, SAT2, and SAT3 are found in pool 6 (Figure 1). Based on prevalence data, serotype and topotype (intra-serotypic variants) distribution, and expert opinions on animal movement, farming systems, and the impact of wildlife, pool 4 can be further divided into the IGAD cluster (Ethiopia, Eritrea, Somalia, Kenya, Uganda, Sudan, and South Sudan) and the East African Community (Burundi, Kenya, Rwanda, Uganda, and the United Republic of Tanzania) [13,29,33].

### 4.2. Distribution and Overall Prevalence of Foot-and-Mouth Disease in the Horn of Africa

FMD is endemic in the Horn of Africa, with variable prevalence in different countries. In Ethiopia, seroprevalence studies using NSP ELISA range from 5.6% to 72.1% in cattle, from 4% to 11% in small ruminants, and 30% in ungulate wildlife [38,39,40,41] in a different corner of the country. In swine, 2% seroprevalence using NSP ELISA was reported [41]. In the wild ungulates, 30% seroprevalence was reported, as reviewed by Abdela [38].

In Kenya, NSP ELISA national seroprevalence was recorded from 52.5% to 93% in different areas of the country [42,43,44] in cattle, whereas in small ruminants [45], 22.5% seroprevalence was documented by NSP ELISA. In swine, the seroprevalence using NSP ELISA was reported to be 54.4% [46], whereas in African buffalo, 77% seroprevalence was reported in Kenya by Omondi et al. [44] using NSP ELISA. In Eritrea, 26% NSP ELISA seroprevalence in cattle was reported [47]. These authors could not find any reports on small ruminants, pigs, or wild animals in Eritrea. In Uganda, herd seroprevalence was 2 to 99% for the randomly sampled herds and 12 to 78% for the purposely sampled herds [48]. The same author documented seroprevalence of FMD in cattle using NSP ELISA to be 65% in seven districts of Uganda [49]. In small ruminants, the documented NSP ELISA seroprevalence was 14% in goats and 22% in sheep [50]. The NSP ELISA seroprevalence of FMD in African buffaloes (*Syncerus caffer*) was recorded to be 74% in Uganda [8]. Solid-phase blocking ELISA-based cattle FMD seroprevalence in Sudan is variable depending on the serotypes and ranges from 3.4% to 49% [5,51]. A seroprevalence of 53.9% using an anti-3ABC antibody ELISA kit in cattle raised together with small ruminants was reported in Sudan [52]. In small ruminants also, a seroprevalence of 14.1% was documented using an anti-3ABC antibody ELISA kit by the same author [52]. In South Sudan, NSP ELISA over all seroprevalence in cattle, sheep, and goats was reported at 37%. Particularly, 56% and 25% NSP ELISA seroprevalence were reported in Unity and Lakes States, respectively [53]. However, these reports are irregular because of an uncoordinated surveillance system in the region. To date, to the best of our knowledge, no extensive research has been performed in countries along the Horn of Africa. Especially in countries such as Eritrea, Somalia, and Djibouti, studies are scarce. This shows that the disease is highly endemic and creates a great socio-economic impact in the region.

### 4.3. Serotype and Topotypes in the Horn of Africa

Four serotypes of FMDV are present in Ethiopia, Kenya, Sudan, and South Sudan (O, A, SAT1, and SAT2) [12], and a fifth serotype has been reported in Uganda from African buffaloes (SAT3) [9]. In Eritrea, serotypes O, A [47], and SAT2 have been reported, and in Somalia and Djibouti, serotype O has been reported as reviewed by Tekleghiorghis and his colleagues [54].

The seven serotypes of FMDV time scale and population dynamics study based on the VP1(1D) region of the genome show an overall mean nucleotide substitution rate of 2.48 × 10^−3^ substitutions/site/year (s/s/yr) and time of origin with a mean age of 432 years. Asia 1 and serotype C, which are not endemic in this region, are also having 6.32 × 10^−3^ and 1.63 × 10^−3^ substitutions/site/year (s/s/yr) over the period of 96 and 82 years, respectively [55]. The presence of a high mutation rate in this virus has generated several topotypes in the last 20 years, among which are the O/EA-4 topotype of serotype O and the SAT1/IX topotypes of serotype SAT1 in Ethiopia [56].

#### 4.3.1. Serotype O Topotypes in the Horn of Africa

**Figure 2:**
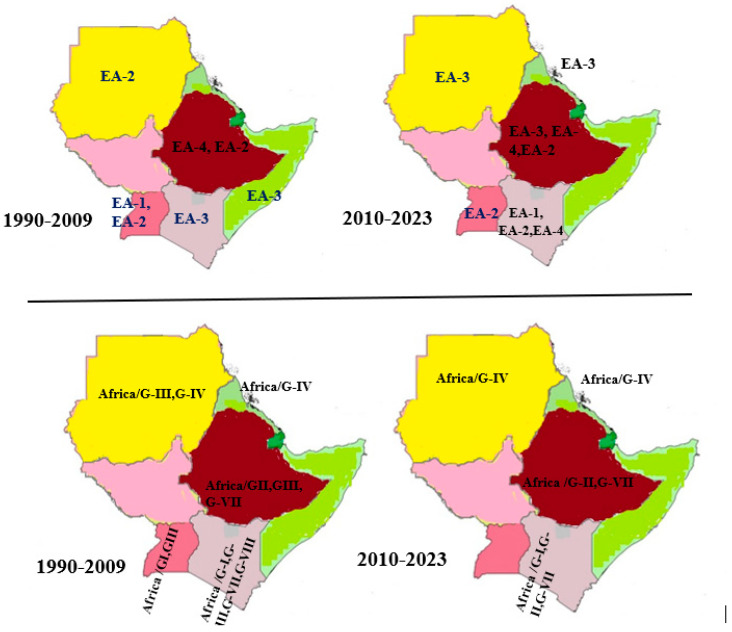
Serotype O (**top panel**) and A (**bottom panel**) topotype distribution and evolution in the Horn of Africa countries (1990–2023).

There are eleven topotypes of serotype O FMDV [57] (and five of them can be found in Sub-Saharan Africa: The most dominant topotype in the Horn of Africa is O/EA-3 which probably originated in Ethiopia and Sudan [58]. Topotype O/EA-3 was first described in Kenya in 1987. Within the Horn of Africa, it was then described in Somalia in 2007, and Ethiopia and Sudan in 2011, respectively. As of 2013, this topotype is known to circulate in Ethiopia, Eritrea, and Sudan [59]. Noteworthy is that FMDV O/EA-3 has spread from the Horn of Africa to the West and further to North-West Africa as well as to North-East Africa and further to the Middle East [60], illustrating the panzootic potential of the O/EA-3 topotype. Topotype O/EA-2 was first described in Sudan in 1999, and within the Horn of Africa, it is now described in Uganda and Kenya since 2007 and 2011, respectively [59,61]; WRLFMD, 2018. In Ethiopia, EA-2 and EA-4 topotypes were circulating from 2008–2014 (Figure 2) [59,62]. Interestingly, O/EA-2 is spreading southward and has occurred in Zambia via Tanzania since 2018 [63] and has spread further to Namibia in 2021 [64] as well as to Malawi and Mozambique in 2022 [65].

In the Horn of Africa, FMDV topotype O/EA-4 was described first in Uganda in 1999 and further spread to Kenya and Ethiopia in 2010 and 2013, respectively [58,59,61], while O/EA-1 was described in Uganda and Kenya in 1996 and 2010, respectively [53,58]. It is considered that serotype O originated in the Horn of Africa, with a higher probability of origination in Kenya and Sudan, which act as a link between East and North Africa. Four clades (VP1 nucleotide sequence differences among topotypes disseminated to different parts of Africa) of serotype O FMDV (EA1,2,3,4) were identified using phylogeographic analysis [66]. For their geographic origin, the first clade was composed of viral sequences from Kenya, Tanzania, and Uganda; the second was in Ethiopia; the third was in Sudan; and the fourth was in West and Central African countries. Using evolutionary parameter estimation, it was found that a mean evolutionary rate of 3.41 × 10^−3^ nucleotide substitutions per site per year over 58 years was recorded (1958–2016) [55,66]. On the other hand, Munsey from Uganda also estimated the VP1 evolutionary rate to be 4.99 × 10^−3^ nucleotide substitutions per site per year [67].

#### 4.3.2. Serotype A Topotypes in the Horn of Africa

Serotype A has three topotypes named Africa, Asia, and Europe-South America (Euro-SA). Within the topotype Africa, there are eight genotypes described. The most dominant genotype in the Horn of Africa was A/Africa genotype III, which was first described in Uganda in 2002 and later described in Ethiopia and Kenya, both in 2005, and in Sudan in 2007 [17]. A/Africa genotype I was also described in Kenya in 2009 and Uganda in 2002, as reviewed by [54], and was recently reported in Kenya by WRLFMD [61]. Serotype A/Africa genotype IV was reported in Sudan and Eritrea in 2006 and 2009, respectively, and is still circulating [61,68,69]. The A/Africa genotype VII was also reported in Kenya and Ethiopia in 2006 and 2009, respectively. This genotype is still circulating in Ethiopia [68]. Genotypes II and VIII were only described in Kenya, as reviewed in [54]. In addition, Lycett et al. recently confirmed serotype A/Africa genotypes I and VII from Northern Africa, with possible spread to the remaining African countries [70]. Another study based on isolates from east Africa confirmed the presence of four genotypes of serotype A (I, II, IV, and VII) [71]. As described by Wekesa et al., [72] genotypes III and VIII were the extinct genotypes from the Horn of Africa (Figure 2).

Lycett et al. estimated the most recent common ancestor of this serotype A as an East African virus dated from 1930 and thought to originate from Eastern Africa (Ethiopia and Kenya) [66]. Using evolutionary parameter estimation, a mean mutation rate of 4.46 × 10^−3^ nucleotide substitutions per site per year was reported by [55,73]. A recent estimate by Xu and Yang revealed a 3.2 × 10^−3^ overall mean nucleotide substitution rate of FMDV serotype A substitution per year per site over 85 years (1932–2017) of evolution. The African topotype means nucleotide substitution rate was estimated at 3.2 × 10^−3^ substitutions per site per year that spans from 1964–2017 [74].

#### 4.3.3. Serotype SAT2 Topotypes in the Horn of Africa

**Figure 3:**
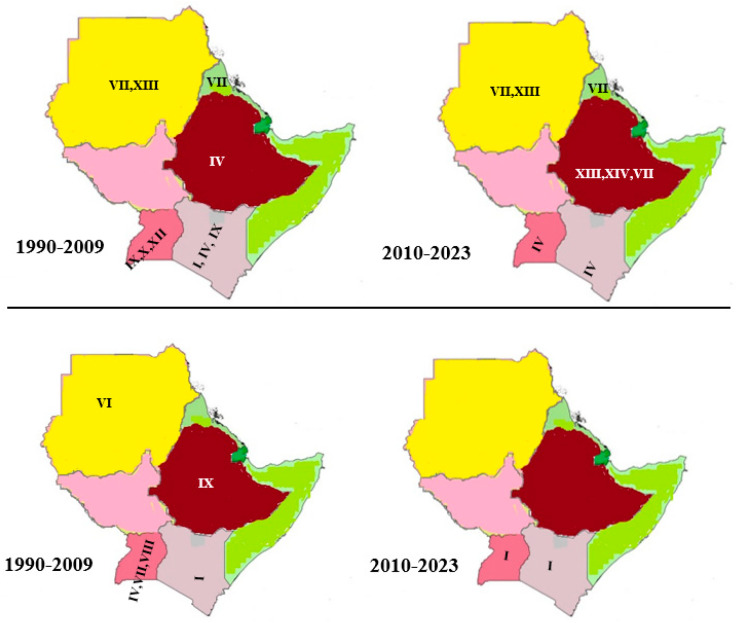
Serotype SAT 2 (**top panel**) and SAT 1 (**bottom panel**) topotype distribution and evolution in the Horn of Africa countries (1990–2023).

The SAT2 serotype has fourteen topotypes (I to XIV) and is mainly limited to Sub-Saharan Africa, with spill-off to the Middle East and Northern Africa crossing the Red Sea and the Sahara Desert [13]. Seven of the fourteen topotypes are found in the Horn of Africa. It is seen that this serotype is more geographically defined than serotypes O and A [7]. The most dominant topotype in the Horn of Africa is SAT2/VII, which was first described in Eritrea in 1998. This topotype was later described in Sudan in 2007, 2010, and 2018, and in Ethiopia in 2009 and 2018 [7,61]. This topotype further spreads to Egypt to the north and is reported as the VII-Alx-12 lineage [69]. Following this, the oldest topotype in the region is topotype SAT2/IV, first described in Ethiopia in 1991 and later in Kenya in 2009, as reviewed by Tekleghiorghis et al. [7]. This topotype spreads to the south of Uganda and back to Kenya in 2016 and 2017, respectively [14,61]. Topotype XIII was reported in Ethiopia and Sudan in 2010 and 2008, respectively [75]. Topotype IX was reported in Uganda and Kenya in 1995 and 1996, as described by Ayelet et al. [12] and reviewed by Tekleghiorghis et al. [7]. Further, topotype XIV was only described in Ethiopia in 1991, and no reports were found in other Horn African countries afterwards (Figure 3).

In contrast to serotypes O and A, SAT2 originates from the southern part of Africa and spreads to the Horn of Africa. There are five geographically defined clades of topotype (I, II, III, IV, and VII) (VP1 nucleotide sequence differences among topotypes) of the SAT2 serotype based on their nucleotide sequence [66]. The first clade (topotype II) is composed of sequences from Botswana, Namibia, and Zimbabwe, whereas the second clade (topotype IV) is from Ethiopians, Kenyans, Ugandans, and Tanzanians. The third clade (topotype I) is from Zimbabwe and all the South African nucleotide sequences. The fourth clade (topotype III) is made of Botswana, Namibia, and Zambia nucleotide sequences. The most diverse clade (topotype VII) of all is the fifth clade, which is composed of Eastern, Western, and Northern African sequences (Cameroon, Egypt, Ethiopia, Libya, Nigeria, and Sudan). Its evolutionary mean mutation rate was estimated to be 1.08 × 10^−3^ nucleotide substitutions per site per year [55,66] for over 67 years, from 1948 to 2015. SAT2 also has a much older common ancestor dating from the early 1700s [70].

#### 4.3.4. Serotype SAT1 Topotypes in the Horn of Africa

For serotype SAT1, thirteen topotypes are reported. Similar to the SAT2 serotype, this serotype is geographically defined as the African continent. The first described topotype of SAT1 was topotype VII, reported in Uganda in the year 1974, and topotype VI in Sudan. Following this, in 1997, topotype VIII was described in Uganda [76]. Topotypes IX and IV were known to circulate in Ethiopia and Uganda in the year 2007 [77]. The recent circulation of SAT1 topotype-I (genetically distinct as compared to the previous isolates of the same topotype) was also confirmed by WRLFMD in 2018 and 2019 in Kenya [69] and Uganda [14]. The most recent report of SAT1 in Kenya was topotype-I, as reported in the year 2021 (Knowles, 2021) (Figure 3). This serotype shows a mean nucleotide substitution rate of 3.59 × 10^−3^ substitutions per site per year [55,66,78] (for over 82 years; 1933–2015).

#### 4.3.5. Serotype SAT3 Topotypes in the Horn of Africa

**Figure 4:**
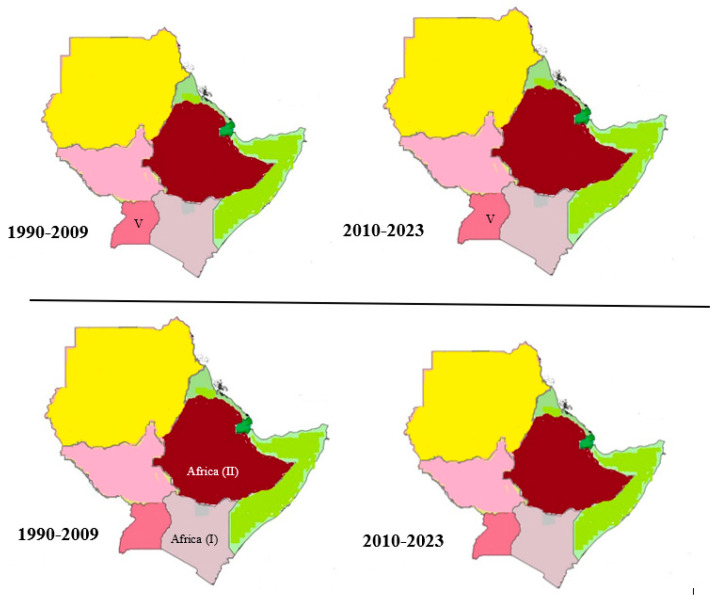
Serotype SAT 3 (**top panel**) and C (**bottom panel**) topotype distribution and evolution in the Horn of Africa countries (1990–2023).

Based on phylogenetic analysis of the VP1 sequence of the FMDV SAT3 serotype, five regionally distinct and geographically defined topotypes of the African continent were identified (I to V). In the Horn of Africa, SAT3 has only been described sporadically by Dhikusooka and his colleagues [76] and reviewed by Tekleghiorghis et al. [54]. Topotype V of this serotype was reported in Uganda in 2013 [76] (Figure 4). This serotype also shows a mean nucleotide substitution rate of 2.58 × 10^−3^ substitutions per site per year over 34 years [55].

#### 4.3.6. Serotype C Topotypes in the Horn of Africa

Serotypes C topotype Africa (I) and (II) were also reported in Kenya and Ethiopia, but they seem extinct from the region (Figure 4). This serotype was reported for the last time in Kenya in 2004 [31,32] (reviewed by Di Nardo et al. [33]; Sebhatu et al. [34] and Rufael et al. [35] detected the serological presence of this serotype in Eritrea and in the Borena zone of southern Ethiopia. No clinical case was reported or detected over the last 15 years for this particular serotype [10].

## 5. The Horn of Africa and the Dynamics of FMD

In the Horn of Africa, four of the seven FMDV serotypes are endemic, and more than 20 topotypes of the more than 60 recognized worldwide are in circulation. In terms of distribution, all countries in the Horn have at least one serotype of FMDV reported. The observed differences in the number of serotypes and topotypes per country might also depend on the capacity of outbreak reporting and investigation in each country. Borders between countries are open to pastoralist herdsmen, for legal and illegal trade, all contributing to livestock movement, including animals infected with FMDV [79,80]. None of the countries in the region have fully implemented the FMD control strategy.

**Figure 5:**
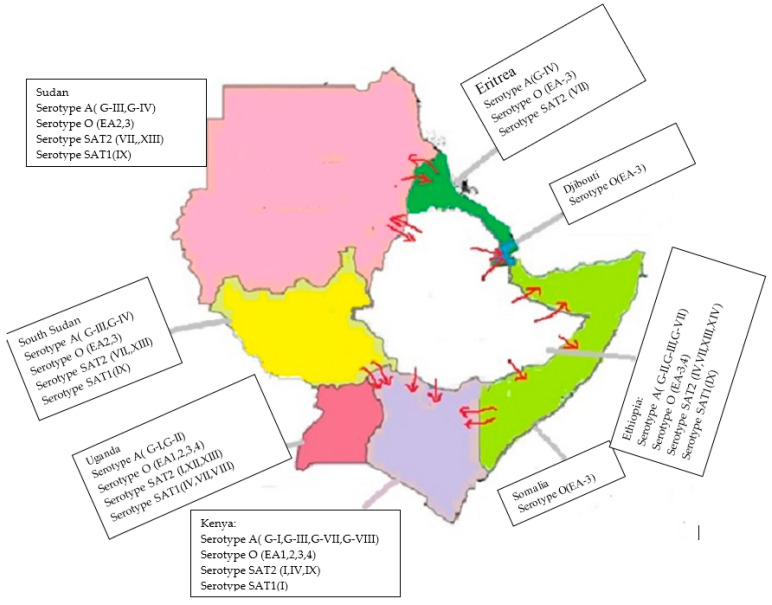
The circulating serotypes and topotypes of the foot-and-mouth disease (FMD) virus in the Horn of Africa. The possible cross-border livestock movement and dynamism of serotypes and topotypes are indicated by arrows.

Among the control/prevention strategies to be used in the region, biosecurity should be taken as a priority considering its thriving results in countries that are declared free [81]. (In the Horn of Africa, vaccination does not always seem to be protective against FMD, not even in the peri-urban semi-intensive production system. The geographical coverage of the data is limited. This needs widespread sampling, isolation, and characterization of the agent in the region to have a broader vaccine antigen that matches the circulating field strain [67,82]. The presence of multiple topotypes, a lack of effective cross-protection, and no control approach all increase the epidemiological burden and dynamism of the disease. Balinda et al. stated the probable cross-border incursion of serotypes EA-3 and EA-4 from Sudan and Ethiopia to Kenya and Tanzania [58]. Ethiopia shares a border with six countries in the region; Kenya and South Sudan share their borders with four countries; Eritrea, Djibouti, Somalia, and Sudan share their borders with three countries, which poses a risk of disease incursion. Therefore, the region is always vulnerable and a hotbed for new topotypes and serotypes incursions/origins. This is especially true where pastoralists share pasture, grazing land, and watering points as well as illegal markets among countries that share a border. For example, this poses a great threat to Ethiopia, given that Kenya has additional topotypes that are not reported in Ethiopia. On Ethiopia’s western front, Sudan and South Sudan are also a threat, with all the risk factors mentioned above holding (Figure 5). Therefore, this region is considered a hotbed of FMD epidemiology, both within it as well as in other parts of the continent and the Middle East [83]. Live animal trade between east Africa and north Africa, the Middle East, Israel, and Palestine in 2017 was believed to facilitate the jump of topotype VII SAT2 FMDV [84]. This jump was recently suspected after the report of SAT2 topotype XIV in Iraq [85].

According to the evidence presented above, FMDV incursions are possible within the Horn of Africa as well as beyond the region, even into neighboring countries. Given that the region is home to four of the seven globally recognized serotypes, coupled with less strict border regulation, the presence of illegal animal trade in this region is a hotbed for FMD outbreaks and poses a threat to other free countries.

## 6. Animal Factor Role in the Epidemiology of FMD

### 6.1. Acute and Persistent Infection

FMD is a disease of ungulate species of domestic and wild origin. Domestic animals (such as cattle, sheep, goats, and pigs) and wild animals (such as African buffalo, deer, and antelope) are naturally susceptible to infection with FMDV. Animals acutely infected with FMDV can show mild, moderate, severe, or no clinical signs. All these animals can transmit the virus to susceptible animals [86]. As reviewed by Stenfeldt and Arzt, cattle, buffalo, and sheep can be persistent carriers of FMDV, whereas pigs can clear the infection within four weeks. In sheep, persistence is not related to whether the disease is clinically manifested or not [87]. Both non-vaccinated (clinically susceptible) and vaccinated (clinically protected) cattle have the chance of becoming persistently infected (carriers) or cleared of infection [87]. From this, it is worthy to pinpoint that the Horn of Africa harbors all domestic and wild ungulates (Table 1) in an FMD endemic setting, though their epidemiological significance is poorly understood.

A recent transmission dynamics study of an active outbreak (acute phase of infection) in cattle in Ethiopia by Tadesse et al. documented animal transmission at a rate of 0.33/day and 0.26/day in crop-livestock mixed farming (CLM) and commercial farming systems, respectively. The same author described the basic reproduction ratio as 1.68 and 1.98, by which a single infected animal can produce another productive infection in CLM and commercial production systems, respectively [89]. Additionally, as reviewed by Paton et al., at least under experimental conditions, transmission of FMDV from acutely infected donor animals to susceptible recipient animals was possible in both cattle and pigs [90]. The same author also stated that the transmission from a carrier animal to a susceptible one is still poorly understood. Bertram et al. also showed that there was no evidence of FMD transmission from a persistently infected animal to naïve calves housed together for six months. This is also true for calves born from carrier animals [91].

Wild animals such as African buffalo found in Kenya, Ethiopia, and Uganda are persistent carriers of FMDV as well as a source of new virus variants [92]. In addition, this animal is considered a primary source of FMDV, particularly the SATs serotypes in the savannah ecosystem. It keeps the virus for up to 400 days but is unlikely to transmit the virus to cattle [93].

Although Arzt et al. have shown that recovered carrier animals can be infectious to naïve animals under certain experimental procedures, they have very low, intermittently detectable amounts of FMDV, implying that domesticated carrier animals are epidemiologically less significant than acutely infected animals [94].

### 6.2. Animal Movement and Trade

In the pastoral environment of Africa, livestock movement is an essential part of daily life to access water sources and grazing pastures, find opportunities for livestock trading, or escape disease or inter-ethnic conflict by crossing borders [79]. The husbandry system practiced varies from no permanent place to live (pastoralist) to a sedentary way of life [95].

Studies in East Africa identified that informal pastoral livestock movements and their product trading influence the spread of human and animal diseases [66,80,96]. A recent study by Munsey and his colleague stated that the dispersal of serotype O FMDV happened as a result of anthropogenic factors [67]. Di Nardo et al. [33] also showed three risks of FMDV dispersal between countries along the Horn of Africa. The defined risks are the border areas between Kenya, Tanzania, and Uganda; the Somali ecosystem (including the Somali region of Ethiopia, Somalia, and the north-eastern region of Kenya); and the bordering areas between East Sudan, northern Ethiopia, and Eritrea. These regions have mutually linked marketing and trade systems. A study by Aman et al., in Ethiopia, also described livestock movement for free grazing during the dry season of the year and local trading of animals during religious festivities as a determinant of seasonal (October to March) FMD outbreaks [97]. Similarly, livestock markets between neighboring districts in Uganda and Tanzania resulted in the occurrence of FMD outbreaks in the two countries; further agent characterization at the genetic level is needed to confirm the relatedness of the two outbreaks [44]. Another study on FMD seroprevalence in the Maasai Mara ecosystem in Kenya pinpointed pastoralist husbandry practices and mixing of different herds at watering points as a risk of infection or spread of FMD [43]. Another study from Tanzania, outside of the Horn of Africa, that used a modeling approach also confirmed that the spread of the pathogen is influenced by the cattle movement network [98].

## 7. Role of Wild Ungulates

Countries from this region, such as Ethiopia, Kenya, and Uganda, are home to African buffalo [99] and susceptible to the foot-and-mouth disease virus, which is potentially transmissible between wild and domestic animals. As reviewed by Gortázar et al. [100], hundreds of animal species can be affected by FMDV either naturally or experimentally. Using a participatory epidemiology approach at the Maasai Mara in Kenya, FMD was identified as one of the livestock diseases at the livestock and wild animal interface [101].

In general, there is a lack of sequence data availability in Africa as a whole and in the Horn of Africa with regards to FMDV circulating in both domestic and wild animals. This affects or limits our understanding of the basic concept of FMD epidemiology in Africa. The role of wildlife ranks highly among the epidemiological determinants of FMD [70]. Research from Kenya by Omondi et al. identified no sequence similarities between SAT1 and SAT2 isolated from African buffalo and sympatric cattle. The same author, on the contrary, described a significant similarity between VP1 FMDV sequences obtained from cattle and African buffalo in other areas of Kenya. This indicates that wild animals might have epidemiological significance [44]. Dhikusooka and his colleagues found that SAT1 FMDV sequences from cattle around Queen Elizabeth National Park in Uganda are different from sequences formerly isolated from the African buffalo. While this strengthens the hypothesis that transmission between wild and domestic animals has not occurred, it also means that this eventuality cannot be ignored [76] and further investigation is recommended as studies in this aspect are scarce [7]. As reviewed by Swanepoel and his colleagues, FMD antigens/antibodies were found in many African ungulates [88]. Most of the wild ungulates on the list are found in the Horn of Africa, but no research data were found regarding FMD prevalence in these animals (Table 1). African buffalo were known to maintain the FMD infection persistently, as described previously [92].

## 8. Vaccination

Systematic control of FMD by vaccination has never been attempted in the Horn of Africa except for sporadic vaccination programs performed by farmers in urban areas who practice a semi-intensive production system. In the pastoralist areas, vaccination against FMD has never been attempted, at least not in Ethiopia [102]. Further, proactive vaccination ahead of the wave of infection in the function of serotype identification would help to reduce the burden [103]. Two producers of FMD vaccine are based in the Horn of Africa: the National Veterinary Institute (NVI) in Debre Zeit (Bishoftu), Ethiopia, and the Kenya Veterinary Vaccines Production Institute (KEVEVAPI), Nairobi, Kenya.

The NVI vaccine is a trivalent containing (O, A, and SAT2), a non-structural protein purified, and the virus is absorbed into concentrated aluminum hydroxide gel [Al(OH)_3_], inactivated with 0.3% of formaldehyde, and adjuvanted with saponin. The duration of protection of this vaccine is a maximum of six months [104]. As reported by Tesfaye and his colleagues, only 10 out of 16 field (EA-3) strains have a good match with the vaccine strain, whereas six of the EA-4 topotypes have a poorer match in Ethiopia [82]. The National Veterinary Institute (NVI), Debre Zeit (Bishoftu), Ethiopia, currently uses O/ETH/38/2005, SAT2/ETH/65/2010, and A/ETH/7/2000 as vaccine strains. The same author also recommended a regular vaccine matching test of the circulating strain with the vaccine strain [105,106].

The FOTIVAX of the KEVEVAPI FMDV vaccine is in the form of an aluminum hydroxide gel [Al(OH)_3_] concentrate that is adjuvanted with saponin [107] and purified with non-structural proteins. It uses the O/KEN/77/78, A/KEN/05/1980, SAT2/K52/84, and SAT1/T1557/71 vaccine strains to formulate a monovalent, bivalent, trivalent, or quadrivalent vaccine depending on the needs of the customer [108]. The FMD vaccine strains used are of historic origin and are less stable as compared to non-African serotypes [109]. This vaccine is prepared with aluminum hydroxide gel and saponin. The O/KEN/77/78 vaccine, commonly used in the region, presents a lower percent antigenic match against the dominant circulating topotypes EA-2 and EA-3 in the region [6]. At least in Kenya, the O/EA-1 topotype is used to formulate the vaccines, resulting in low cross-protection with circulating viruses [64]. For serotype, FMDV vaccine matching studies suggest the reformulation of commercial vaccines that are currently used in the region and comprise A-KEN-05-1980 and A-ETH-06-2000 antigens [49,51,71,89].

The KEVEVAPI duration of protection is 6 months, or every 4 months for better protection (https://kevevapi.or.ke/fotivax/, accessed on 20 February 2023). However, in the Horn of Africa, vaccination does not always seem to be protective against FMD, not even in the peri-urban semi-intensive production system. Maintaining a sound biosecurity practice helps to prevent the introduction and spread of the FMDV into a particular farm or country [108].

As reviewed by Ambaye Kenubih, the importance of both structural and non-structural proteins of the FMDV on both the cellular and humoral arms of the immune system should be studied for sterile and long-lasting immunity development [107]. As reviewed recently by a project working in East Africa on FMD control, vaccination is considered the major control approach, but each virus pool in the endemic area should have a more specific or tailored vaccine for that specific region or pool of topotypes [110].

## 9. Conclusions

Foot and mouth disease epidemiology in the Horn of Africa is complex as a result of serotype and topotype diversity. In addition to this, unrestricted animal movements and insignificant control approaches exacerbate the epidemiological dynamics of the disease. The Horn of Africa harbors five FMDV serotypes and more than twenty topotypes, with a continuous threat of the spread of new topotypes within the region. Countries such as Ethiopia, South Sudan, Sudan, and Kenya are known as hotbeds for the emergence of new FMDV strains in the region and in other regions as well. The livestock movement in the area within and between the regions is unrestricted due to diverse factors, of which the search for markets, watering points, and pastures is the most important. The presence of wild animals is the third major factor affecting the epidemiology of the disease. However, the presence of African buffalo in the Horn of Africa may have less of an effect on FMD infections in cattle in comparison to the southern part of Africa. The danger of FMD in the region is increasing because of high genetic diversity, poor husbandry, a poor control approach, no biosecurity practices coupled with unregulated trade, and pastoral herds movement in search of pasture and water as a result of recurring drought. To this end, early diagnosis and implementation of a suitable control approach need to focus on biosecurity.

## Figures and Tables

**Table 1:** List of wild ungulate species present in the Horn of Africa countries that are susceptible to FMDV infection, according to Swanepoel et al. [88].

Name of Wild Ungulates	Ethiopia	Kenya	Sudan	Eritrea	Djibouti	Uganda	South Sudan	Somalia
Buffalo, African/Cape	yes	yes	yes	-	-	yes	yes	yes
Elephant, African	yes	yes	-	-	-	yes	yes	
Bushpig	yes	yes	yes	-	-	yes	yes	yes
Grant’s Gazelle	yes	yes	-	-	-	yes	yes	-
Gazelle	yes	yes	-	-	-	-	-	yes
Kudu	yes	yes	yes		yes	yes	yes	yes
Giraffe	yes	yes	yes	yes	-	yes	yes	yes
Oribi	yes	yes	yes	-	-	-	-	-
Reedbuck, Bohor	yes	yes	yes			yes	yes	
Roan antelope	yes	yes	yes	-	-	-	-	-
Impala	-	yes	-	-	-	yes	-	-
warthog	yes	yes	yes	-	-	yes	-	-
Günther’s dik-dik,	yes	yes	yes	yes		yes		
Gazella	yes		yes	yes	yes	-	yes	-
Salt’s dik-dik	-	yes	yes	-	yes	-	yes	yes

Source: https://www.ncbi.nlm.nih.gov/pmc/articles/PMC7912165/, accessed on 12 March 2022.

## Data Availability

The data will be provided by the corresponding author upon request.

## References

[1] Alary V., Bourzat D. (2008). Global Animal Products’ Supply and Demand-Challenges of the Horn of Africa. https://agritrop.cirad.fr/548607/1/document_548607.pdf.

[2] Current Availability and Future Requirements of Livestock Feeds. https://epashupalan.com/10961/animal-husbandry/current-availability-and-future-requirements-of-livestock-feeds/.

[3] The IGAD Regional Peste des Petits Ruminants (PPR) Progressive Control and Eradication Strategy. https://rr-africa.woah.org/wp-content/uploads/2019/09/progressive-control-and-eradication-strategy-1.pdf.

[4] Horn of Africa Drought: Regional Humanitarian Overview & Call to Action (Revised 21 September 2022). https://fscluster.org/news/horn-africa-drought-regional#:~:text=Over%208.9%20million%20livestock%E2%80%94which,latest%20FSNWG%20Drought%20Special%20Report.

[5] Alexandersen S., Mowat N. (2005). Foot-and-mouth disease: Host range and pathogenesis. Curr. Top. Microbiol. Immunol..

[6] Lloyd-Jones K., Mahapatra M., Upadhyaya S., Paton D.J., Babu A., Hutchings G., Parida S. (2017). Genetic and antigenic characterization of serotype O FMD viruses from East Africa for the selection of suitable vaccine strain. Vaccine.

[7] Tekleghiorghis T., Moormann R.J.M., Weerdmeester K., Dekker A. (2016). Foot-and-mouth Disease Transmission in Africa: Implications for Control, a Review. Transbound. Emerg. Dis..

[8] Ayebazibwe C., Mwiine F.N., Tjørnehøj K., Balinda S.N., Muwanika V.B., Okurut A.R.A., Belsham G.J., Normann P., Siegismund H.R., Alexandersen S. (2010). The role of African buffalos (syncerus caffer) in the maintenance of foot-and-mouth disease in Uganda. BMC Vet. Res..

[9] Dhikusooka M.T., Tjørnehøj K., Ayebazibwe C., Namatovu A., Ruhweza S., Siegismund H.R., Wekesa S.N., Normann P., Belsham G.J. (2015). Foot-and-mouth disease virus serotype SAT 3 in long-horned ankole Calf, Uganda. Emerg. Infect. Dis..

[10] Paton D.J., Di Nardo A., Knowles N.J., Wadsworth J., Pituco E.M., Cosivi O., Rivera A.M., Kassimi L.B., Brocchi E., de Clercq K. (2021). The history of foot-and-mouth disease virus serotype C: The first known extinct serotype?. Virus Evol..

[11] Foot and Mouth Disease. https://www.woah.org/en/disease/foot-and-mouth-disease/.

[12] Ayelet G., Mahapatra M., Gelaye E., Egziabher B.G., Rufeal T., Sahle M., Ferris N.P., Wadsworth J., Hutchings G.H., Knowles N.J. (2009). Genetic characterization of foot-and-mouth disease viruses, Ethiopia, 1981–2007. Emerg. Infect. Dis..

[13] Rweyemamu M., Roeder P., MacKay D., Sumption K., Brownlie J., Leforban Y., Valarcher J.F., Knowles N.J., Saraiva V. (2008). Epidemiological patterns of foot-and-mouth disease worldwide. Transbound. Emerg. Dis..

[14] WRLFMD Foot-and-Mouth Disease. Quarterly Report January to March 2020. https://www.wrlfmd.org/sites/world/files/quick_media/WRLMEG-2020-00003B-UGA-GTR-O-SAT2.pdf.

[15] Megersa B., Beyene B., Abunna F., Regassa A., Amenu K., Rufael T. (2009). Risk factors for foot and mouth disease seroprevalence in indigenous cattle in Southern Ethiopia: The effect of production system. Trop. Anim. Health Prod..

[16] Wright C.F., Morelli M.J., Thébaud G., Knowles N.J., Herzyk P., Paton D.J., Haydon D.T., King D.P. (2011). Beyond the consensus: Dissecting within-host viral population diversity of foot-and-mouth disease virus by using next-generation genome sequencing. J. Virol..

[17] Ferretti L., Pérez-Martín E., Zhang F., Maree F., de Klerk-Lorist L.M., van Schalkwykc L., Juleff N.D., Charleston B., Ribeca P. (2020). Pervasive within-host recombination and epistasis as major determinants of the molecular evolution of the foot-and-mouth disease virus capsid. Phil. Trans. R. Soc. B.

[18] Ferretti L., Di Nardo A., Singer B., Lasecka-Dykes L., Logan G., Wright C.F., Pérez-Martín E., King D.P., Tuthill T.J., Ribeca P. (2018). Within-Host Recombination in the Foot-and-Mouth Disease Virus Genome. Viruses.

[19] Jamal S.M., Ferrari G., Ahmed S., Normann P., Belsham G.J. (2011). Molecular characterization of serotype Asia-1 foot-and-mouth disease viruses in Pakistan and Afghanistan; emergence of a new genetic Group and evidence for a novel recombinant virus. Infect. Genet. Evol..

[20] Ahmed N.H., Osman N.A., Alfouz W., Saeed H.M. (2021). Serological detection and genetic characterization of foot-and-mouth disease virus from cattle in northern sudan, 2016–2018. Vet. Anim. Sci..

[21] Domingo E., Soria M.E., Gallego I., de Ávila A.I., García-Crespo C., Martínez-González B., Gómez J., Briones C., Gregori J., Quer J. (2020). A new implication of quasispecies dynamics: Broad virus diversification in absence of external perturbations. J. Mol. Epidemiol. Evol. Genet. Infect. Dis..

[22] Li C., Shi J., Wang H., Rivera-Serrano E.E., Yang D., Zhou G., Sun C., Cameron C.E., Yu L. (2020). Polymerase fidelity contributes to Foot-and-Mouth Disease Virus pathogenicity and transmissibility in vivo. J. Virol..

[23] Domingo E., Granoff A., Webster R.G. (1999). Quasispecies. Encyclopedia of Virology.

[24] Domingo E., Escarmı C., Baranowski E., Ruiz-Jarabo C.M., Carrillo E., Núñez J.I., Sobrino F. (2003). Evolution of foot-and-mouth disease virus. Virus Res..

[25] Arzt J., Fish I.H., Bertram M.R., Smoliga G.R., Hartwig E.J., Pauszek S.J., Holinka-Patterson L., Segundo F.C.D.-S., Sitt T., Rieder E. (2021). Simultaneous and Staggered Foot-and-Mouth Disease Virus Coinfection of Cattle. J. Virol..

[26] Brito B., Pauszek S.J., Hartwig E.J., Smoliga G.R., Vu L.T., Dong P.V., Stenfeldt C., Rodriguez L.L., King D.P., Knowles N.J. (2018). A traditional evolutionary history of foot-and-mouth disease viruses in Southeast Asia challenged by analyses of non-structural protein coding sequences. Sci. Rep..

[27] Rweyemamu M.M., Astudillo V.M. (2002). Global perspective for foot and mouth disease control. Rev. Sci. Tech..

[28] Samuel A.R., Knowles N.J. (2001). Foot-and-mouth disease type O viruses exhibit genitically and geographically distinct evolutionary lineages (topotypes). J. Gen. Virol..

[29] Knowles N.J., Samuel A.R. (2003). Molecular epidemiology of foot-and-mouth disease virus. Virus Res..

[30] Paton D.J., Sumption K.J., Charleston B. (2009). Options for control of foot-and-mouth disease: Knowledge, capability and policy. Philos. Trans. R. Soc. B Biol. Sci..

[31] Knowles N.J., Swabey K.G., Midgley R.J., Davies P.R., Wadsworth J. Global molecular epidemiology of foot-and-mouth disease virus type C. Proceedings of the 14th Meeting of the European Study Group on the Molecular Biology of Picornavirus.

[32] Roeder P.L., Knowles N.J. (2008). Foot-and-mouth disease virus type C situation: The first target for eradication?. Report of the Session of the Research Group of the Standing Technical Committee of EUFMD.

[33] Di Nardo A., Knowles N.J., Paton D.J. (2011). Combining livestock trade patterns with phylogenetics to help understand the spread of foot and mouth disease in sub-Saharan Africa, the Middle East and Southeast Asia. Rev. Sci. Tech..

[34] Sebhatu T.T. Foot-and-Mouth Disease Sero-Surveillance in Africa and Vaccine Matching. https://dspace.library.uu.nl/handle/1874/299019.

[35] Rufael T., Catley A., Bogale A., Sahle M., Shiferaw Y. (2008). Foot and mouth disease in the Borana pastoral system, southern Ethiopia and implications for livelihoods and international trade. Trop. Anim. Health Prod..

[36] Dubie T., Negash W. (2021). Seroprevalence of bovine foot and mouth disease (FMD) and its associated risk factors in selected districts of Afar region, Ethiopia. Vet. Med. Sci..

[37] Freimanis G.L., Di Nardo A., Bankowska K., King D.J., Wadsworth J., Knowles N.J., King D.P. (2016). Genomics and outbreaks: Foot and mouth disease. Rev. Sci. Tech..

[38] Abdela N. (2017). Sero-prevalence, risk factors and distribution of foot and mouth disease in Ethiopia. Acta Trop..

[39] Mesfine M., Nigatu S., Belayneh N., Jemberu W.T. (2019). Sero-Epidemiology of Foot and Mouth Disease in Domestic Ruminants in Amhara Region, Ethiopia. Front. Vet. Sci..

[40] Sulayeman M., Dawo F., Mammo B., Gizaw D., Shegu D. (2018). Isolation, molecular characterization and sero-prevalence study of foot-and-mouth disease virus circulating in central Ethiopia. BMC Vet. Res..

[41] Woldemariyam F.T., De Vleeschauwer A., Hundessa N., Muluneh A. (2022). Risk Factor Assessment, Sero-Prevalence, and Genotyping of the Virus that Causes Foot-and-Mouth Disease on Commercial Farms in Ethiopia from October 2018 to February 2020. Agriculture.

[42] Kibore B., Gitao C.G., Sangula A., Kitala P. (2014). Foot and mouth disease sero-prevalence in cattle in Kenya. J. Vet. Med. Anim. Health.

[43] Nthiwa D., Bett B., Odongo D., Kenya E., Wainaina M., Grazioli S., Foglia E., Brocchi E., Alonso S. (2020). Seroprevalence of foot-and-mouth disease virus in cattle herds raised in Maasai Mara ecosystem in Kenya. Prev. Vet. Med..

[44] Omondi G.P., Gakuya F., Arzt J., Sangula A., Hartwig E., Pauszek S., Smoliga G., Brito B., Perez A., Obanda V. (2020). The role of African buffalo in the epidemiology of foot-and-mouth disease in sympatric cattle and buffalo populations in Kenya. Transbound. Emerg. Dis..

[45] Chepkwony E.C., Gitao G.C., Muchemi G.M., Sangula A.K., Kairu-Wanyoike S.W. (2021). Epidemiological study on foot-and-mouth disease in small ruminants: Sero-prevalence and risk factor assessment in Kenya. PLoS ONE.

[46] Kibore B., Gitao C.G., Sangula A., Kitala P. (2014). Porcine FMD Sero-prevalence in Kenya and its potential effect. Am. J. Res. Commun..

[47] Tekleghiorghis T., Weerdmeester K., van Hemert-Kluitenberg F., Moormann R.J.M., Dekker A. (2017). Foot-and-Mouth Disease Seroprevalence in Cattle in Eritrea. Transbound. Emerg. Dis..

[48] Mwiine F.N., Velazquez-Salinas L., Ahmed Z., Ochwo S., Munsey A., Kenney M., Lutwama J.J., Maree F.F., Lobel L., Perez A.M. (2019). Serological and phylogenetic characterization of foot and mouth disease viruses from Uganda during cross-sectional surveillance study in cattle between 2014 and 2017. Transbound. Emerg. Dis..

[49] Mwiine F.N., Ayebazibwe C., Alexandersen S., Olaho-Mukani W., Ademun A.R.O., Tjørnehøj K. Serotype Specificity of Antibodies against Foot-and-Mouth Disease Virus in Cattle in Selected Districts in Uganda. https://academicjournals.org/journal/JVMAH/article-full-text-pdf/1776E601433.

[50] Ur-Rehman S., Arshad M., Hussain I., Iqbal Z. (2014). Detection and Seroprevalence of Foot and Mouth Disease in Sheep and Goats in Punjab, Pakistan. Transbound. Emerg. Dis..

[51] Alfouz W., Raouf Y.A., Ahmed N.H., Hamid A.E., Osman N.A. (2021). Sero-epidemiology of foot-and-mouth disease in Darfur area, Western Sudan. Vet. Res. Commun..

[52] Yousif H., Almutlab A.A., Hassen A.A., Al-Majali A., Tibbo M. (2017). Role of small ruminants in the epidemiology of foot-and-mouth disease in Sudan. Bull. Anim. Health Prod. Afr..

[53] Ochi E., Ismail A.O. (2014). A Review on Epidemiology of Foot and Mouth Disease (FMD) in South Sudan. Rep. Opin..

[54] Tekleghiorghis T., Moormann R.J.M., Weerdmeester K., Dekker A. (2014). Serological evidence of foot-and-mouth disease virus serotype C & SAT-1 infections in Eritrea. Transbound. Emerg. Dis..

[55] Tully D.C., Fares M.A. (2008). The tale of a modern animal plague: Tracing the evolutionary history and determining the time-scale for foot and mouth disease virus. Virology.

[56] Gizaw D., Tesfaye Y., Wood B.A., Di Nardo A., Shegu D., Muluneh A., Bilata T., Belayneh R., Fentie A., Asgdome H. (2020). Molecular characterization of foot-and-mouth disease viruses circulating in Ethiopia between 2008 and 2019. Transbound. Emerg. Dis..

[57] Kerfua S.D., Shirima G., Kusiluka L., Ayebazibwe C., Martin E., Arinaitwe E., Cleaveland S., Haydon D.T. (2019). Low topotype diversity of recent foot-and-mouth disease vrius serotypes O and A from districts located along the Uganda and Tanzania border. J. Vet. Sci..

[58] Balinda S.N., Sangula A.K., Heller R., Muwanika V.B., Belsham G.J., Masembe C., Siegismund H.R. (2010). Diversity and transboundary mobility of serotype O foot-and-mouth disease virus in East Africa: Implications for vaccination policies. Infect. Genet. Evol. J. Mol. Epidemiol. Evol. Genet. Infect. Dis..

[59] WRLFMD Foot-and-Mouth Disease Report 2013. https://www.wrlfmd.org/sites/world/files/quick_media/OIE-FAO%20FMD%20Ref%20Lab%20Report%20October-December%202013.pdf.

[60] Canini L., Blaise-Boisseau S., Nardo A.D., Shaw A.E., Romey A., Relmy A., Bernelin-Cottet C., Salomez A.-L., Haegeman A., Ularamu H. (2022). Identification of diffusion routes of O/EA-3 topotype of foot-and-mouth disease virus in Africa and Western Asia between 1974 and 2019—A phylogeographic analysis. Transbound. Emerg. Dis..

[61] WRLFMD Foot-and-Mouth Disease Quarterly Report April to June 2018. https://www.wrlfmd.org/sites/world/files/quick_media/OIE-FAO%20FMD%20Ref%20Lab%20Report%20Apr-Jun%202018.pdf.

[62] Negusssie H., Kyule M.N., Yami M. (2011). Outbreak investigations and genetic characterization of foot-and-mouth disease virus in Ethiopia in 2008/2009. Trop. Anim. Health Prod..

[63] Banda F., Sinkala Y., Mataa L., Lebea P., Sikombe T., Kangwa H.L., Fana E.M., Mokopasetso M., Wadsworth J., Knowles N.J. (2021). Characterization of Foot-and-Mouth Disease Viruses in Zambia-Implications for the Epidemiology of the Disease in Southern Africa. Viruses.

[64] Banda F., Shilongo A., Hikufe E.H., Khaiseb S., Kabajani J., Shikongo B., Set P., Kapapero J.K., Shoombe K.K., Zaire G. (2022). The first detection of a serotype O foot-and-mouth disease virus in Namibia. Transbound. Emerg. Dis..

[65] Foot-and-Mouth Disease. https://www.fao.org/3/cc4292en/cc4292en.pdf.

[66] Florian Duchatel M.B. (2018). Circulation of Foot-and-Mouth Disease Virus in Africa and identification of the underlying constraints using Phylogeographic methods. bioRxiv.

[67] Munsey A., Norbert F., Sylvester M., Salinas L.V., Ahmed Z., Maree F., Rodriguez L.L., Rieder E., Perez A., Dellicour S. (2021). Phylogeographic analysis of foot-and mouth disease virus serotype O dispersal and associated drivers in East Africa. Mol. Ecol..

[68] WRLFMD Foot-and-Mouth Disease Quarterly Report October to December 2018. https://www.wrlfmd.org/sites/world/files/quick_media/OIE-FAO%20FMD%20Ref%20Lab%20Report%20Oct-%20Dec%202018.pdf.

[69] WRLFMD Foot-and-Mouth Disease Quarterly Report July to September 2018. https://www.wrlfmd.org/sites/world/files/WRLFMD-2018-00020-SUD-GTR-O-SAT2_001.pdf.

[70] Lycett S., Tanya V.N., Hall M., King D.P., Mazeri S., Mioulet V., Knowles N.J., Wadsworth J., Bachanek-Bankowska K., Ngu Ngwa V. (2019). The evolution and phylodynamics of serotype A and SAT2 foot-and-mouth disease viruses in endemic regions of Africa. Sci. Rep..

[71] Bari F.D., Parida S., Tekleghiorghis T., Dekker A., Sangula A., Reeve R., Haydon D.T., Paton D.J., Mahapatra M. (2014). Genetic and antigenic characterisation of serotype A FMD viruses from East Africa to select new vaccine strains. Vaccine.

[72] Wekesa S.N., Sangula A.K., Belsham G.J., Muwanika V.B., Heller R., Balinda S.N., Masembe C., Siegismund H.R. (2014). Infection, Genetics and Evolution Genetic diversity of serotype A foot-and-mouth disease viruses in Kenya from 1964 to 2013; implications for control strategies in eastern Africa. Infect. Genet. Evol..

[73] Duchatel F., Bronsvoort B.M., Lycett S. (2019). Phylogeographic Analysis and Identification of Factors Impacting the Diffusion of Foot-and-Mouth Disease Virus in Africa. Front. Ecol. Evol..

[74] Xu W., Yang M. (2021). Genetic variation and evolution of foot-and-mouth disease virus serotype A in relation to vaccine matching. Vaccine.

[75] WRLFMD Foot-and-Mouth Disease. Quarterly Report January to December 2020. https://www.fao.org/eufmd/resources/reports/gmr/fr/.

[76] Dhikusooka M.T., Ayebazibwe C., Namatovu A., Belsham G.J., Siegismund H.R., Wekesa S.N., Balinda S.N., Muwanika V.B., Tjørnehøj K. (2016). Unrecognized circulation of SAT 1 foot-and-mouth disease virus in cattle herds around Queen Elizabeth National Park in Uganda. BMC Vet. Res..

[77] Balinda S.N., Tjørnehøj K., Muwanika V.B., Sangula A.K., Mwiine F.N., Ayebazibwe C., Masembe H., Siegismund R., Alexandersen S. (2009). Prevalence estimates of antibodies towards foot-and-mouth disease virus in small ruminants in Uganda. Transbound. Emerg. Dis. Dec..

[78] Sangula A.K., Belsham G.J., Muwanika V.B., Heller R., Balinda S.N., Masembe C., Siegismund H.R. (2010). Evolutionary analysis of foot-and-mouth disease virus serotype SAT 1 isolates from east africa suggests two independent introductions from southern africa. BMC Evol. Biol..

[79] Bouslikhane M. (2015). Cross border movements of animals and animal products and their relevance to the epidemiology of animal diseases in Africa. Africa OIE Reg. Comm..

[80] Grace D., Little P. (2020). Informal trade in livestock and livestock products. Rev. Sci. Tech..

[81] Biosecurity. https://hro.house.texas.gov/focus/biosecure.pdf.

[82] Munsey A., Mwiine F.N., Ochwo S., Velazquez-Salinas L., Ahmed Z., Maree F., Rodriguez L.L., Rieder E., Perez A., VanderWaal K. (2019). Spatial distribution and risk factors for foot and mouth disease virus in Uganda: Opportunities for strategic surveillance. Prev. Vet. Med..

[83] Hekal S.H.A., Al-Gaabary M.H., El-Sayed M.M., Sobhy H.M., Fayed A.A.A. (2019). Seroprevalence of some Infectious transboundry diseases in cattle imported from Sudan to Egypt. J. Adv. Vet. Anim. Res..

[84] Wubshet A.K., Dai J., Li Q., Zhang J. (2019). Review on outbreak dynamics, the endemic serotypes, and diversified topotypic profiles of foot andmouth disease virus isolates in Ethiopia from 2008 to 2018. Viruses.

[85] FAO Prioritization of Antigen Management with International Surveillance. https://www.fao.org/3/cb1799en/cb1799en.pdf.

[86] Robinson L., Knight-Jones T.J.D., Charleston B., Rodriguez L.L., Gay C.G., Sumption K.J., Vosloo W. (2016). Global Foot-and-Mouth Disease Research Update and Gap Analysis: 7—Pathogenesis and Molecular Biology. Transbound. Emerg. Dis..

[87] Stenfeldt C., Arzt J. (2020). The Carrier Conundrum; A Review of Recent Advances and Persistent Gaps Regarding the Carrier State of Foot-and-Mouth Disease Virus. Pathogens.

[88] Swanepoel H., Crafford J., Quan M. (2021). A Scoping Review of Viral Diseases in African Ungulates. Vet. Sci..

[89] Tadesse B., Molla W., Mengsitu A., Jemberu W.T. (2019). Transmission dynamics of foot and mouth disease in selected outbreak areas of northwest Ethiopia. Epidemiol. Infect..

[90] Paton D.J., Gubbins S., King D.P. (2018). Understanding the transmission of foot-and-mouth disease virus at different scales. Curr. Opin. Virol..

[91] Bertram M.R., Vu L.T., Pauszek S.J., Brito B.P., Hartwig E.J., Smoliga G.R., Hoang B.H., Phuong N.T., Stenfeldt C., Fish I.H. (2018). Lack of Transmission of Foot-and-Mouth Disease Virus from Persistently Infected Cattle to Naïve Cattle under Field Conditions in Vietnam. Front. Vet. Sci..

[92] Cortey M., Ferretti L., Pérez-Martín E., Zhang F., de Klerk-Lorist L.-M., Scott K., Freimanis G., Seago J., Ribeca P., van Schalkwyk L. (2019). Persistent Infection of African Buffalo (*Syncerus caffer*) with Foot-and-Mouth Disease Virus: Limited Viral Evolution and No Evidence of Antibody Neutralization Escape. J. Virol..

[93] Maree F., de Klerk-Lorist L.-M., Gubbins S., Zhang F., Seago J., Pérez-Martín E., Reid L., Scott K., van Schalkwyk L., Bengis R. (2016). Differential Persistence of Foot-and-Mouth Disease Virus in African Buffalo Is Related to Virus Virulence. J. Virol..

[94] Arzt J., Belsham G.J., Lohse L., Bøtner A., Stenfeldt C. (2018). Transmission of Foot-and-Mouth Disease from Persistently Infected Carrier Cattle to Naive Cattle via Transfer of Oropharyngeal Fluid. mSphere.

[95] Robinson T.P., Thornton P.K., Francesconi G.N., Kruska R.L., Chiozza F., Notenbaert A.M., Cecchi G., Herrero M.T., Epprecht M., Fritz S. (2011). Global Livestock Production Systems.

[96] Duchatel F. (2021). Virus Phylogeography at the Wild/Domestic Animal Interface. Ph.D. Thesis.

[97] Aman E., Molla W., Gebreegizabher Z., Jemberu W.T. (2020). Spatial and temporal distribution of foot and mouth disease outbreaks in Amhara region of Ethiopia in the period 1999 to 2016. BMC Vet. Res..

[98] Chaters G.L., Johnson P.C.D., Cleaveland S., Crispell J., De Glanville W.A., Doherty T., Matthews L., Mohr S., Nyasebwa O.M., Rossi G. (2019). Analysing livestock network data for infectious disease control: An argument for routine data collection in emerging economies. Philos. Trans. R. Soc. Biol. Sci..

[99] Cornélis D., Melletti M., Korte L., Ryan S.J., Mirabile M., Prin T., Prins H.H. (2014). African buffalo Syncerus caffer (Sparrman, 1779). Ecology, Evolution and Behaviour of Wild Cattle: Implications for Conservation.

[100] Gortázar C., Barroso P., Nova R., Cáceres G. (2022). The role of wildlife in the epidemiology and control of Foot-and-mouth-disease And Similar Transboundary (FAST) animal diseases: A review. Transbound. Emerg. Dis..

[101] Nthiwa D., Alonso S., Odongo D., Kenya E., Bett B. (2019). A participatory epidemiological study of major cattle diseases amongst Maasai pastoralists living in wildlife-livestock interfaces in Maasai Mara, Kenya. Trop. Anim. Health Prod..

[102] Jemberu W.T., Molla W., Dagnew T., Rushton J., Hogeveen H. (2020). Farmers’ willingness to pay for foot and mouth disease vaccine in different cattle production systems in Amhara region of Ethiopia. PLoS ONE.

[103] Casey-Bryars M., Reeve R., Bastola U., Knowles N.J., Auty H., Bachanek-Bankowska K., Fowler V.L., Fyumagwa R., Kazwala R., Kibona T. (2018). Waves of endemic foot-and-mouth disease in eastern Africa suggest feasibility of proactive vaccination approaches. Nat. Ecol. Evol..

[104] Foot and Mouth Disease (FMD) Vaccine Description. https://www.nvi.com.et/products/vaccines-against/ruminant-and-equine-diseases/fmd-2/.

[105] Tesfaye Y., Khan F., Gelaye E. (2021). Vaccine matching and antigenic variability of foot-and-mouth disease virus serotypes O and A from 2018 Ethiopian isolates. Int. Microbiol..

[106] Tesfaye Y., Khan F., Yami M., Wadsworth J., Knowles N.J., King D.P., Gelaye E. (2020). A vaccine-matching assessment of different genetic variants of serotype O foot-and-mouth disease virus isolated in Ethiopia between 2011 and 2014. Arch. Virol..

[107] Kenubih A. (2021). Foot and Mouth Disease Vaccine Development and Challenges in Inducing Long-Lasting Immunity: Trends and Current Perspectives. Vet. Med. Res. Rep..

[108] Sangula A.K., Siegismund H.R., Belsham G.J., Balinda S.N., Masembe C., Muwanika V.B. (2011). Low diversity of foot-and-mouth disease serotype C virus in Kenya: Evidence for probable vaccine strain re-introductions in the field. Epidemiol. Infect..

[109] Jackson B., Harvey Y., Perez-Martin E., Wilsden G., Juleff N., Charleston B., Seago J. (2021). The selection of naturally stable candidate foot-and-mouth disease virus vaccine strains for East Africa. Vaccine.

[110] Hammond J.M., Maulidi B., Henning N. (2021). Targeted FMD Vaccines for Eastern Africa: The AgResults Foot and Mouth Disease Vaccine Challenge Project. Viruses.

